# Effects of adjunctive light-activated disinfection and probiotics on clinical and microbiological parameters in periodontal treatment: a randomized, controlled, clinical pilot study

**DOI:** 10.1007/s00784-020-03727-1

**Published:** 2021-02-04

**Authors:** Michael Patyna, Vicky Ehlers, Benjamin Bahlmann, Adrian Kasaj

**Affiliations:** Department of Periodontology and Operative Dentistry, University Medical Center of the Johannes Gutenberg-University Mainz, Augustusplatz 2, 55131 Mainz, Germany

**Keywords:** Periodontitis, Subgingival debridement, Adjunctive periodontal treatment, Light-activated disinfection, Antibacterial photodynamic therapy, Probiotics

## Abstract

**Objectives:**

The aim of this pilot study was to evaluate the clinical and microbiological outcomes of light-activated disinfection (LAD) alone or combined with probiotics as an adjunct to non-surgical periodontal treatment.

**Materials and methods:**

In this single-blinded, randomized, controlled clinical pilot study, 48 patients (28 females and 20 males) with untreated periodontitis (stages II and III, grade B) were included. Using a parallel-group design, patients were randomly assigned into 3 groups to receive subgingival debridement (SD) alone (group 1, *n* = 16), SD with LAD (group 2, *n* = 16), or SD with LAD plus probiotic treatment (group 3, *n* = 16). Probing pocket depth (PPD), clinical attachment level (CAL), bleeding on probing (BOP), gingiva-index simplified (GIs), plaque-control record (PCR), and subgingival microbiological samples were analyzed at baseline, 3 months, and 6 months of follow-up.

**Results:**

All treatment modalities demonstrated clinical improvements in PPD and CAL at 6 months compared to baseline but without a statistical significant difference between the groups. The combination of SD + LAD + probiotic treatment (group 3) demonstrated significantly greater reductions in BOP, GIs, and red complex bacteria *P. gingivalis* and *T. forsythia* compared with other groups at 6 months (*p* < 0.05).

**Conclusions:**

A single application of LAD as an adjunct to SD provided no additional clinical and microbiological benefits compared to SD alone. The combination of SD + LAD + probiotic treatment in group 3 led to further improvements of the inflammatory parameters.

**Clinical relevance:**

The additional use of probiotics in periodontal treatment can be a useful approach to support inflammation and infection control of periodontal tissues. Further studies are necessary to determine the extent of added benefit for this treatment approach.

## Introduction

Nowadays, periodontitis can be considered the result of interaction between microbial factors and host immune response [1]. It is an imbalance within the immune system, and as such, both bacteria and environmental factors play an important role in the development and manifestation of this disease [2, 3]. The primary goal in periodontal treatment is the reduction or suppression of pathogens from periodontal sites [4]. This can be achieved through subgingival debridement (SD) using machine-driven or hand instruments [5–8].

To support and further improve the clinical and microbiological outcomes of subgingival instrumentation, adjunctive antimicrobial therapies have been proposed. Among them, systemic or topical antimicrobials are most commonly used [9–11]. However, the worldwide increase in antibiotic resistance has become a major concern. Thus, resistance of various pathogens against antibiotics poses an increasing threat to medicine, one that is linked to the efficacy of medication in general. Microbial resistance towards antibiotics has also been linked to the subgingival microbiota [12–14]. Therefore, it seems reasonable to consider potential alternatives to the use of antibiotics in periodontal therapy.

Antimicrobial photodynamic therapy (aPDT) has been proposed as an adjunct to non-surgical periodontal treatment. In aPDT, a specific photosensitizer binds to the target bacteria and gets activated by light of appropriate wavelength. This activation generates mainly singlet oxygen, which is toxic to bacteria [15–17]. Synthetic and semi-synthetic dyes such as methylene blue, toluidine blue, acridine orange, benzoporphyrin derivatives, erythrosine, and azulene can be used as photosensitizing agents [18]. Thus, destruction of the outer membrane of *Porphyromonas gingivalis* without harming host cells was observed when toluidine blue alone was used in a concentration of 12.5 g/ml [19–21]. The light used for aPDT can be provided by laser systems or by non-coherent light sources such as light-emitting diodes (LED). LED devices as a light source are more compact, flexible, and less expensive compared to traditional lasers [22]. The LED-based photodynamic therapy is often referred to as photoactivated disinfection (PAD) or light-activated disinfection (LAD). Although there is growing evidence for the use of aPDT as an adjunct to conventional non-surgical treatment of periodontitis, the potential clinical benefit of this treatment approach remains controversial [23]. Moreover, there is only limited evidence on the clinical relevance of LED-based aPDT when used in conjunction with non-surgical periodontal therapy [8].

More recently, the use of probiotics has been advocated as another beneficial adjunct to SD in non-surgical treatment and management of periodontitis. In 1965, the term “probiotics” was first introduced as an opposite meaning to the term antibiotics by Lilly and Still-Well. It was described as a substance that promotes the growth of other organisms [24]. Traditionally, probiotics have been used to treat gastrointestinal diseases and are usually composed of the genera *Lactobacillus* or *Bifidobacteria* [25]. Studies demonstrated that oral administration of probiotics can alter the bacterial population of supra- and subgingival plaque [26, 27]. Probiotics may show the ability to adhere to oral tissues and can be tolerant to fluctuations and changes in the oral environment [28]. Although several studies looked at the clinical and microbiological effects of aPDT or probiotics associated with non-surgical periodontal therapy, little is known about the combination of both treatment strategies [8, 29–34]. Therefore, the aim of the present pilot study was to evaluate the clinical and microbiological outcomes of LAD alone or combined with probiotics as an adjunct to SD in non-surgical periodontal therapy.

## Materials and methods

### Study population

The study was conducted between January 2016 and October 2017. Forty-eight systemically healthy patients (mean age 58.3 ± 2 years; 20 males, 28 females) were enrolled from the Periodontal Clinic of the University. The study protocol was reviewed and approved by the Ethics Committee of Rhineland-Palatinate, Germany (Protocol 837.375.15 (10143)) and followed the Declaration of Helsinki for experimentation involving humans. This trial was registered at the German Register of Clinical Studies (DRKS; ID: DRKS00023158). This pilot study was designed and conducted to test the feasibility of applying LED and probiotics as an adjunct to non-surgical periodontal therapy.

**Fig. 1:**
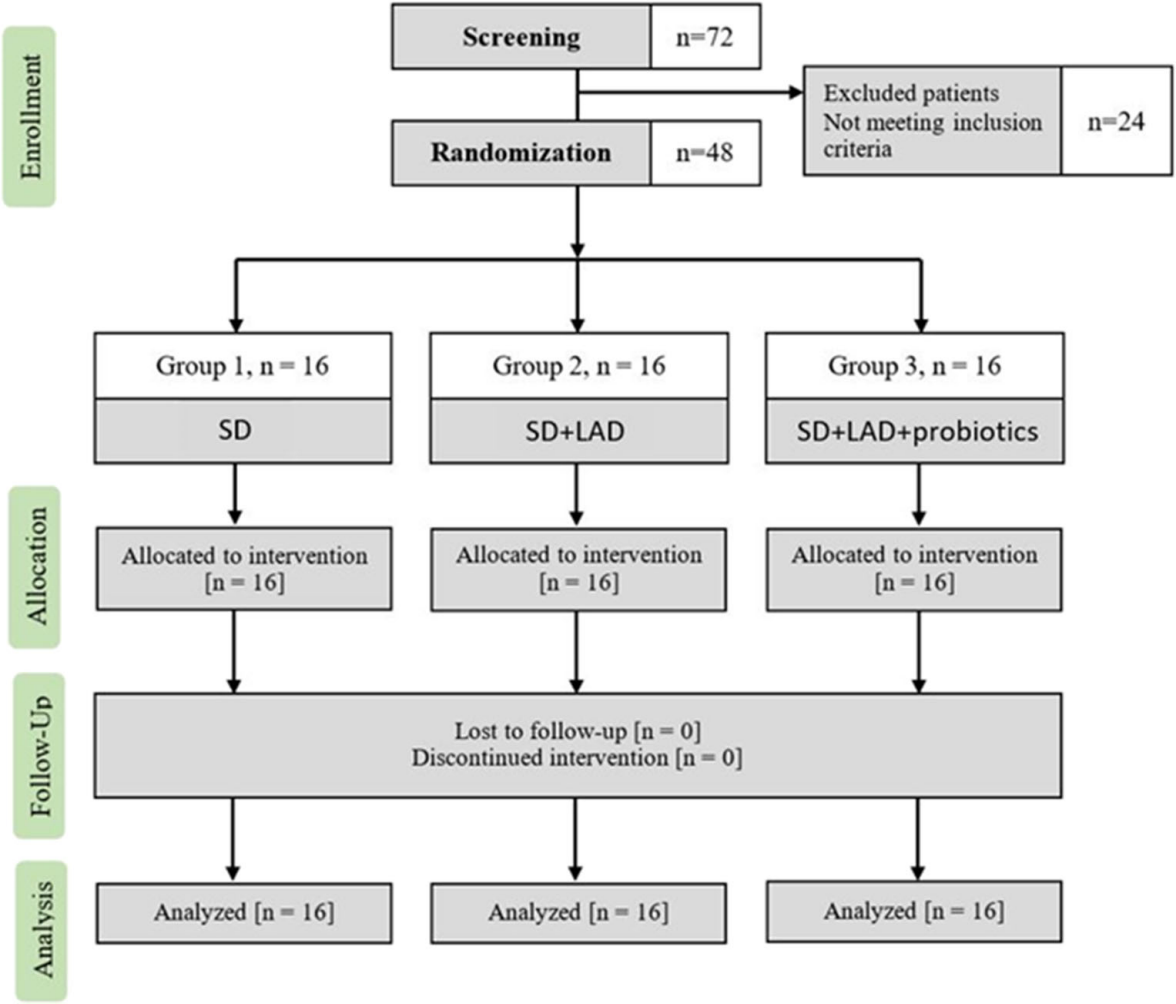
Study flowchart

The inclusion criteria for the study were the diagnosis of periodontitis stage II or III and grade B [35], the presence of at least six sites with probing pocket depth (PPD) ≥ 5 mm, bleeding on probing (BOP), and at least 20 remaining teeth. The exclusion criteria were diabetes mellitus, HIV, heart disease, osteoporosis, or lactose intolerance. Further exclusion criteria included a positive history of periodontal or antibiotic treatment in the previous 6 months, or any pharmaceutical treatment that could influence the treatment outcome, smoking and pregnancy. All patients received oral and written explanation of the purpose of the study and signed an informed consent. A flowchart to illustrate the study design is presented in Fig. 1.

### Sample size calculation and treatment allocation

The required sample size was calculated based on the primary outcome measures using a stand-alone power analysis program. The significance level was set to 2.5%. An 80% power was chosen to identify a significant difference in mean PPD reduction of 1 mm between groups; this corresponds to an effect size of *f*^2^ = 0.27. To detect an effect size of *f*^2^ = 0.27, 16 patients per group were required.

An investigator not involved in the clinical trial created the allocation sequence by a computer-generated list to generate random numbers for 16 subjects per treatment group (3 groups). The use of sealed non-transparent envelopes ensured allocation concealment.

### Treatment protocol

The present study was designed as a randomized, controlled, single-blinded, parallel-group pilot study. All patients received full-mouth supragingival scaling and oral hygiene instructions. Subgingival mechanical debridement was performed at all sites with PPD > 3 mm by using an ultrasonic device (EMS, Electro Medical Systems S.A., Nyon, Switzerland) and hand instruments (Hu-Friedy, Chicago, IL, USA) under local anesthesia (Ultracain® D-S 1:200.000, Sanofi-Aventis, Germany). The debridement was carried out in one appointment without any time restrictions until the operator considered the tooth surfaces to be adequately debrided and planed. The treatment was performed by a trained and standardized periodontist who was not informed about the treatment allocation.

Following the completion of instrumentation, the randomization envelope was opened, and patients were assigned to the three investigated treatment groups.

In group 1, patients received mechanical debridement alone. In group 2 (SD + LAD), after controlling bleeding by using gauze swabs and/or sponge pellets followed by repeated irrigation and air-drying, a photosensitizer (Toluidine blue O, Fotosan Agent®, CMS Dental ApS, Copenhagen, Denmark) was applied additionally in the periodontal pockets using a flexible applicator tip and left for 60 s. Subsequently, a LED device (Fotosan®630, CMS Dental ApS, Copenhagen, Denmark) with a wavelength of 628 nm (2000–4000 mW/cm^2^) was used subgingivally to irradiate the photosensitizer for 10 s at each side of the tooth. After completion of the LAD procedure, each periodontal pocket was rinsed with saline solution to remove the photosensitizer. Group 3 (SD + LAD + probiotics) received the same treatment as group 2 (SD + LAD). Besides, probiotics were administered locally and systemically containing *Lactobacillus brevis* 7480 CECT and *Lactobacillus plantarum* 7481 CECT (ProlacSan®, CMS Dental ApS, Copenhagen, Denmark). Thus, following SD and LAD treatment, a probiotic gel (ProlacSan® Gel 1.2 ml, 6 × 10^9^ CFU) was applied subgingivally to fill the periodontal pocket. Moreover, subjects were instructed to take one oral probiotic lozenges with a daily dose of 62.5 mg *L. brevis* and 62.5 mg *L. plantarum* (ProlacSan® lozenges, 1.2 × 10^9^ CFU) per day for 3 months.

### Clinical parameters

The clinical parameters were assessed at baseline and at 3 and 6 months following treatment. All measurements were performed by one experienced and calibrated examiner who was masked and unaware of the treatment protocol. The calibration of the examiner was ensured by selecting ten patients who were not included in the study. Each patient had at least two pairs of contralateral molars with a probing depth of ≥ 5 mm. The patients were examined twice within 48 h. If the two measurements coincided identically in 90% of the teeth examined, the examiner was considered to be calibrated [36].

Following clinical parameters were recorded:

1. *Gingiva-Index simplified* (GIs, Lindhe 1983) was recorded at the beginning of each session to assess the gingival status.
2. *Plaque-Control Record* (PCR, O’Leary 1972) was scored to assess the oral hygiene status of the patients at each session.
3. *Probing pocket depth* (PPD) was measured from the free gingival margin to the bottom of the periodontal pocket.
4. *Clinical attachment level* (CAL) was measured from the cemento-enamel junction to the base of the pocket.
5. *Bleeding on probing* (BOP) was scored based on the presence or absence of bleeding within 30 s after probing.

PPD, CAL, and BOP were measured at six sites per tooth (mesio-buccal, buccal, disto-buccal, disto-lingual, lingual, and mesio-lingual) using a conventional periodontal probe (PCP-UNC 15, Hu-Friedy®, Chicago, IL, USA).

### Microbiological evaluation

Subgingival plaque samples were collected at baseline, 3 months, and 6 months following treatment from the deepest pocket of each quadrant. Before sampling, the test sites were dried by air and isolated with cotton rolls. Subgingival samples were collected with sterile paper points inserted into the selected sites for a time period of 10 s [37]. The samples were transferred to a sterile vial (pooled sample) and sent to a commercial laboratory for analysis. The analysis was performed with a commercially available DNA probe kit (IAI Pado Test, Institute of Applied Immunology, IAI, Zuchwil, Switzerland) to identify the following periopathogenic microorganisms: *Aggregatibacter actinomycetemcomitans (Aa)*, *Tannerella forsythia (Tf)*, *Porphyromonas gingivalis (Pg)*, and *Treponema denticola (Td)*. The test had a detection threshold of 10^3^ for *A. actinomycetemcomitans* and 10^4^ for all other bacteria [38].

### Statistical analysis

Statistical analysis was performed with the statistics program SPSS (IBM Corp. 2013. IBM SPSS Statistics for Windows, Version 23.0, Armonk, New York, USA). Unit of analysis in all statistical tests was the individual. Mean and standard deviations of clinical and microbial variables were calculated for each time point. The primary outcome measures were differences between the groups for changes in PPD after 3 and 6 months compared to baseline. Secondary outcome measures included CAL, BOP, GIs, PCR, and counts of the bacterial species. After testing the normality of data, a repeated-measures ANOVA for the statistical evaluation of the clinical and microbiological parameters’ differences from baseline to 3 and 6 months was used. A *p* value < 0.05 was considered to indicate statistical significance.

## Results

All patients successfully completed the study. The postoperative healing was uneventful in all cases, and no adverse events or complications were recorded during the study. Patient demographic characteristics at baseline are shown in Table 1.

**Table 1:** Patient demographic characteristics at baseline

	Group 1	Group 2	Group 3
*n*	16	16	16
Age in years mean (±SD)	58.38 (± 14.65)	59.63 (± 13.23)	57.06 (± 12.88)
Gender (female/male)	7/9	11/5	10/6

*n*, number of patients; *SD*, standard deviation

### Clinical outcomes

The means and standard deviations of the clinical parameters (PPD, CAL) at baseline and at 3 and 6 months after treatment are presented in Table 2**.** Baseline examination demonstrated that the three study groups showed similar characteristics for clinical parameters PPD and CAL. All treatment modalities resulted in improvements of PPD and CAL at 6 months compared with baseline (Table 2). The intergroup comparison of PPD and CAL showed no significant difference between treatment groups at 3 and 6 months. Secondary clinical parameters (BOP, GIs, PCR) improved in all three groups (Table 3). At 3 and 6 months, a significantly greater reduction of BOP was observed in group 3 when compared to the other treatment groups (*p* < 0.05; Table 3). Concerning GI values, there was a statistically significant reduction in group 3 when compared to group 2 at 6 months (*p* < 0.05; Table 3). PCR showed no significant differences between the groups at 3 and 6 months following treatment.

**Table 2:** Mean values (±SD) of PPD and CAL in the treatment groups at baseline, 3 months, and 6 months

Groups	Baseline	3 months	*p* value Δ (0–3 months)	6 months	*p* value Δ (0–6 months)
PPD (mm ± SD)					
1	4.23 (± 0.76)	3.43 (± 0.45)	< 0.01	3.45 (± 0.43)	< 0.01
2	3.97 (± 0.16)	3.40 (± 0.13)	< 0.01	3.19 (± 0.08)	< 0.001
3	4.71 (± 0.19)	4.06 (± 0.23)	n.s.	3.69 (± 0.26)	< 0.01
CAL (mm ± SD)					
1	5.95 (± 1.12)	5.09 (± 0.77)	< 0.05	5.17 (± 0.82)	n.s.
2	5.37 (± 0.80)	4.85 (± 0.78)	n.s.	4.74 (± 0.71)	n.s.
3	6.81 (± 1.28)	6.11 (± 1.53)	n.s.	5.80 (± 1.79)	n.s.

*PPD*, probing pocket depth; *CAL*, clinical attachment level; “Δ” difference for each group between baseline and 3 or 6 months; *SD*, standard deviation; *n.s.*, no significant difference

**Table 3:** Mean values (±SD) of BOP, GIs, and PCR in the treatment groups at baseline, 3 months, and 6 months

Groups	Baseline	3 months	*p* value Δ (0–3 months)	6 months	*p* value Δ (0–6 months)
BOP (% ± SD)					
1	19.06 (± 13.02)	9.88 (± 9.63)*	n.s.	11.31 (± 12.15)*	n.s.
2	19.81 (± 9.43)	12.81 (± 10.84)*	n.s.	9.88 (± 9.59)*	< 0.05
3	34.00 (± 25.30)	12.13 (± 9.14)	< 0.001	4.88 (± 6.72)	< 0.001
GIs (% ± SD)					
1	19.12 (± 13.03)	8.84 (± 6.87)	< 0.01	6.10 (± 4.53)	< 0.001
2	15.06 (± 10.97)	6.82 (± 8.01)	< 0.05	5.87 (± 6.73)*	< 0.05
3	29.09 (± 25.12)	11.50 (± 15.13)	< 0.05	3.18 (± 5.33)	< 0.001
PCR (% ± SD)					
1	27.24 (± 26.18)	11.09 (± 9.87)	< 0.01	7.00 (± 6.43)	< 0.01
2	11.91 (± 7.96)	5.41 (± 5.44)	< 0.05	4.77 (± 6.01)	< 0.05
3	19.85 (± 14.60)	10.64 (± 10.50)	n.s.	4.48 (± 6.28)	< 0.001

*BOP*, bleeding on probing; *GIs*, gingiva-index simplified; *PCR*, plaque-control record; “Δ” difference for each group between baseline and 3 or 6 months; *SD*, standard deviation; *n.s.*, no significant difference

*p < 0.05, significantly different to group 3

### Microbiological outcomes

All patients in this trial showed high counts of red complex bacteria at baseline (Table 4). Since only a few patients were tested positive for *A. actinomycetemcomitans*, statistical analysis for this pathogen was excluded. In group 3 (SD + LAD + probiotics), a significantly higher reduction in *P. gingivalis* (*p* < 0.01) and *T. forsythia* (*p* < 0.05) was observed at 6 months when compared to the other treatment groups (Table 4). Apart from that, there were no significant differences among treatments with respect to the mean counts of the tested species at any time point.

**Table 4:** Mean counts of four periopathogenic bacteria at baseline, 3 months, and 6 months

Groups	Cell counts × 10^6^ (±SD)		Cell counts × 10^6^ (±SD)	*p* value Δ (0–6 months)
	Baseline	3 months	6 months	
*Tannerella forsythia*				
1	3.43 (± 2.64)	2.55 (± 2.72)	2.65 (± 1.87)*	n.s.
2	2.68 (± 2.37)	2.63 (± 2.60)	2.86 (± 2.50)*	n.s.
3	4.98 (± 2.96)	3.79 (± 2.53)	2.60 (± 2.07)	< 0.05
*Porphyromonas gingivalis*				
1	3.55 (± 4.33)	2.40 (± 2.63)	2.11 (± 1.92)*	n.s.
2	4.27 (± 3.44)	3.90 (± 4.77)	3.41 (± 3.55)*	n.s.
3	9.27 (± 6.73)	5.30 (± 3.98)	3.59 (± 3.32)	< 0.01
*Treponema denticola*				
1	1.20 (± 1.44)	1.10 (± 1.32)	0.83 (± 0.91)	n.s.
2	1.08 (± 1.11)	0.98 (± 1.54)	1.53 (± 1.87)	n.s.
3	2.37 (± 2.14)	1.44 (± 2.04)	1.70 (± 1.87)	n.s.

“Δ” difference for each group between baseline and 6 months; *SD*, standard deviation; *n.s.*, no significant difference

*p < 0.05, significantly different to group 3

## Discussion

The aim of the present randomized controlled clinical pilot study was to evaluate the clinical and microbiological effects of LAD in combination with probiotics as an adjunctive therapy to non-surgical periodontal treatment and to compare the results with those obtained after SD + LAD and SD alone. Analysis of the data showed that the SD + LAD + probiotics protocol resulted in improvements in some of the evaluated clinical parameters. In addition, this treatment approach was able to show further reductions in levels of *P. gingivalis* and *T. forsythia* in subgingival plaque samples. However, the hypothesis that the adjunctive use of probiotics would further improve clinical outcomes of SD + LAD was only partially confirmed in this study. Moreover, the study was not able to confirm an additional benefit for LAD compared to traditional SD. Thus, all three treatment approaches resulted in similar improvements of PPD and CAL values with no significant differences between the groups. This observation is in agreement with previous studies that found no additional clinical benefit of single aPDT application in conjunction with conventional SD in comparison to SD alone [30, 39–42]. Similar clinical results were obtained when LED instead of laser diodes was used as the light source in aPDT [43]. A recent study also failed to demonstrate an additional clinical benefit for the combination of SD + PDT + probiotics compared to SD alone [30]. Therefore, current scientific data on the effectiveness of photodynamic therapy seem to be controversial with no clear clinical guidelines for the clinicians [44, 45]. This may be attributed, at least in part, to the high diversity of light-emitting devices (lasers, LED system) and photosensitizers [46, 47]. Thus, it has been demonstrated that the energy doses released from a laser device can potentially affect the antimicrobial efficacy of PDT treatment [48]. On the other hand, the efficacy of each photosensitizer is influenced by various factors, such as solubility, administration technique, retention time, stability, excitation wavelength, biocompatibility, and clearance rate [49]. Taken together, the optimization of aPDT procedures requires the optimal choice of photosensitizer dose, duration of light application, as well as the time span between photosensitizer application and light exposure.

The present study showed that the benefits of the combination approach with SD + LAD + probiotics were more evident in terms of BOP and GI reductions. It is reasonable to assume that the observed additional effect for BOP and GIs was associated with the adjunctive probiotic protocol, since no greater reductions in GIs and BOP were found for SD + LAD compared to SD alone. Indeed, previous studies were unable to confirm an additional effect for LED-based PDT over conventional SD with respect to clinical inflammatory parameters [43, 50]. In further support of this finding, Giannopoulou et al. (2012) showed that application of PDT was not able to further reduce cytokine and acute-phase protein levels in gingival crevicular fluid when compared with traditional SD [51].

The positive effects of the adjunct probiotic-therapy on clinical inflammatory parameters (BOP, GIs) observed in the present study may be explained by the ability of probiotics to modulate the host’s microbiota [52]. Indeed, several studies reported that probiotics are able to inhibit the growth of periodontopathogenic bacteria, decrease the level of proinflammatory cytokines, and improve periodontal clinical parameters [52–56]. Our findings are further corroborated by previous studies showing that the administration of probiotics as an adjunct to SD provides a positive impact on clinical parameters of gingival inflammation [57, 58]. Similarly, a recent systematic review found a significant BOP reduction (− 14.66%) for SD + probiotic treatment versus SD alone at short-term [27].

Concerning the microbiological parameters in the present study, a significant decrease of red complex bacteria was observed with SD + LAD + probiotics when compared with the other groups at 6 months. These results are in line with other clinical studies that reported a significant reduction in red complex bacteria following the use of adjunctive probiotics [58, 59]. Montero et al. (2017) reported that the adjunctive use of probiotics promoted a significant microbiological impact by reducing the count of *T. forsythia*, which further supports our results [60]. However, in this study, a higher baseline percentage of BOP in group 3 compared to groups 1 and 2 was found. Also, the microbiological analysis showed a higher baseline proportion of *T. forsythia* and *P. gingivalis* in group 3. Nevertheless, the potential for allocation bias was considered small as the investigator had no knowledge of the patients other than the necessary details required for randomization. Therefore, baseline imbalances occurred by chance rather than allocation bias. The magnitude of chance imbalance was not rated as clinically significant [61, 62]. If only the average values of the BOP in all groups after 3 months are taken into consideration, the data appear to be balanced. Consequently, the inflammatory and microbiological parameters in group 3 improved after 6 months compared to groups 1 and 2 and could demonstrate the effect of periodontal therapy with probiotics. The microbiological benefits might be explained by probiotics’ ability to delay the re-colonization of periodontal pockets by periodontal pathogens. Thus, Tekce et al. (2015) observed that in patients treated with SD and probiotics re-colonization with anaerobic microorganisms was slowed for up to 6 months, which is also consistent with our findings [59]. However, several other mechanisms of action have been proposed for probiotics, including modulation of host response, production of antibacterial substance, competitive exclusion, competition for essential nutrients, and enhancement of mucosal barrier function [63]. In this context, it has also to be considered that different strains of probiotics are available as an adjunct in periodontal treatment and that these can provide different benefits. Among them, the probiotic species most commonly used to treat periodontal disease belong to the genera *Lactobacillus* or *Bifidobacterium* [27]. In the present study, probiotics containing the common strains *L. brevis* and *L. plantarum* were employed.

The differences in terms of probiotic strains, mode of administration, and duration of use may limit direct comparison across studies. Thus, patients in the present study took the lozenges for 3 months following the manufacturer’s protocol (ProlacSan®, CMS Dental ApS, Copenhagen, Denmark). The dosage and frequency that would allow the best clinical outcome are still unclear, which is one of the main limitations of this study. We also do not know which results could be obtained with other administration modalities. Another limitation of the study is the 6-month follow-up period.

In principle, this randomized controlled clinical pilot study did not aim to demonstrate and prove a specific non-inferiority or superiority hypothesis but rather to test the feasibility of using PDT and probiotics as an adjunctive therapy to non-surgical periodontal treatment. Therefore, future studies may be planned to evaluate the non-inferiority or superiority of adjunctive use of LAD and probiotics compared to SD alone.

## Conclusions

All three investigated treatment modalities resulted in PPD and CAL improvements at 6 months compared to baseline but without a significant difference between the groups. A single application of LAD as an adjunct to SD provided no additional clinical and microbiological benefits compared to SD alone. The combination of SD + LAD + probiotics did not lead to significant improvements in PPD and CAL when compared to SD + LAD and SD alone. Despite the known limitations of the study, the adjunctive use of LAD + probiotics may represent a valuable non-invasive adjunct to reduce the inflammatory parameters.
